# Magnetic Molecularly Imprinted Polymer Combined with Solid-Phase Extraction for Purification of *Schisandra chinensis* Lignans

**DOI:** 10.3390/polym16223124

**Published:** 2024-11-08

**Authors:** Huijuan Xu, Lihan Sun, Yufei Du, Wenxin Duan, Wei Li, Sha Luo, Bing Liang, Chunhui Ma, Gaofeng Pan

**Affiliations:** 1 Key Laboratory of Bio-Based Material Science and Technology, Ministry of Education, College of Material Science and Engineering, Northeast Forestry University, Harbin 150040, China; xuhj0323@163.com (H.X.); sunlihan@163.com (L.S.); duyufei@163.com (Y.D.); duanwenxin@163.com (W.D.); liwei19820927@126.com (W.L.); luo.sha.85@163.com (S.L.); 2 Mudanjiang Heng Feng Paper Co., Ltd., Mudanjiang 157013, China; liangbing@163.com

**Keywords:** *Schisandra chinensis* (Turcz.) Baill. (*S. chinensis*), magnetic, molecularly imprinted polymers (MIPs), solid-phase extraction (SPE), lignans

## Abstract

Molecularly imprinted polymers (MIPs) can specifically recognize template molecules in solution with imprinted cavities. Due to their capacity for scalable production, they can be used to isolate target products from natural products for industrial production in the fields of pharmaceuticals and food. In this study, magnetic single-template molecularly imprinted polymers (St-MIPs) instead of magnetic multi-template molecularly imprinted polymers (Mt-MIPs) were prepared by surface imprinting using Schizandrol A as a template molecule and deep eutectic solvent (DES) as a functional monomer, combined with solid-phase extraction (SPE) for the adsorption and separation of Schizandrol A, Schisantherin A, Schizandrin A, and Schizandrin B from *Schisandra chinensis* (Turcz.) Baill. (*S. chinensis*) fruits extracts. The synthesized MIPs were characterized by FT-IR, TEM, SEM, TG, XRD and VSM, and their adsorption properties were also evaluated. MIPs can specifically recognize the template molecules with high reusability. The purity of the total *S. chinensis* lignans after SPE was 74.05%, among which that of Schizandrol A, Schisantherin A, Schizandrin A, and Schizandrin B was 33.38%, 8.69%, 16.33% and 15.67%, respectively. Moreover, the one-step synthesis of carrier was easy to operate. And St-MIPs reduced the production cost compared with Mt-MIPs. This study provides a new idea for natural product separation by molecular imprinting technology (MIT).

## 1. Introduction

*Schisandra chinensis* (Turcz.) Baill. (*S. chinensis*) belongs to the broad family of magnolia plants. *S. chinensis* has a long history of medical use as a tonic and astringent agent in China, Korea and Japan [1]. Studies have indicated that the major bioactive components of *S. chinensis* are free dibenzocyclooctadiene lignans [2,3]. Among them, the ones with the highest content are Schizandrol A, Schisantherin A, Schizandrin A and Schizandrin B. These compounds have higher medicinal potential as they protect the cardiovascular and cerebrovascular systems, enhance cardiovascular and cerebrovascular activity, regulate the nervous system, reduce transaminases, and have anti-allergy properties, among other effects [4,5,6]. Because of the complex composition of natural products and the relatively low content of target products, traditional extraction methods exhibit lower extraction yield and purity because of the complex composition of natural products and the relatively low content of target products. Hence, the pursuit of new isolation materials for efficient enrichment is of considerable research interest.

The molecular imprinting technique (MIT) is a technique that is based upon the self-assembly polymerization of a template molecule with polymerizable monomers bearing functional groups, which are capable of interacting with the template molecule (functional monomers), followed by polymerization [7]. Firstly, the template molecules are pre-polymerized with functional monomers and cross-linkers in a solution under the influence of an initiator, in which the template molecules interact with the functional monomers to form template monomer complexes. Then, the initiator acts in the reaction system to carry out a hinge polymerization reaction on the cross-linking agent around the previously formed complexes, producing a highly cross-linked three-dimensional network polymer. After the completion of the polymerization reaction, the template molecules in the polymer are removed using a suitable solvent, and a hollow structure with a three-dimensional lattice structure is obtained at the blotting site. The geometry of the hollow and the positions of the internal functional groups are complementary to those of the template molecules used, and the bonding mechanism is similar to that of a “lock-and-key”, with different template molecules corresponding to the blotted hollow. The corresponding imprinted cavities of different template molecules cannot recognize other molecules. Compared with other recognition systems, molecularly imprinted polymers (MIPs) have the advantages of good selectivity, re-usability, being inexpensive and entailing simple preparation, and having the ability to be produced in large quantities for ease of industrial production [8,9]. Wang synthesized a hydrophilic imprinted polymer to identify and detect sulindac in sewage with extremely high selectivity and adsorption capacity of above 95% after eight cycles [10]. In recent years, MIPs have been used in molecular recognition and detection in the fields of environmental analysis [11], biomarkers [12], pathogen detection [13], synthesis of explosives [14], pharmaceutical analysis [15], drug delivery [16], food safety [17], and sensors [18]. Solid-phase extraction (SPE) is a separation and purification method based on the theory of liquid chromatography. SPE has the advantages of high enrichment factor, high recovery, rapid phase separation, low cost, low consumption of organic solvents, and the capacity of combination with different detection techniques in on-line or off-line modes [19]. However, it also lacks specificity. In contrast, molecular imprint SPE uses a highly selective MIP as an adsorbent, which can effectively remove matrix interference and improve the accuracy and sensitivity of the analysis [20,21,22,23]. Hence, the inclusion of MIP in SPE holds promise to improve selectivity during the separation of lignans from *S. chinensis*.

In MIPs, functional monomers are polymerizable monomers with double bonds or characteristic functional groups, which can interact with template molecules. Deep eutectic solvent (DES), a novel type of green extraction solvent with low volatility, thermal stability and tunability, in addition to being non-toxic, biodegradable, less expensive and easily prepared, can be structurally adjusted according to one’s needs [24,25,26,27,28]. Therefore, when DES is used as a functional monomer, the number of functional groups can be increased according to the needs of the reaction by choosing suitable hydrogen bond acceptors (HBAs) and hydrogen bond donors (HBDs) so as to better bind with the template molecules and improve the imprinting ability of MIPs [29,30].

In this study, MIPs were prepared using *S. chinensis* lignans as the template, magnetic ferric oxide-wrapped modified silica as the carrier, and DES as the functional monomer. It should be noted that four *S. chinensis* lignans have the similar parent nucleus, differing only with a small number of functional groups. Single-template molecularly imprinted polymers (St-MIPs) only required one kind of template molecule to identify four *S. chinensis* lignans with similar parent nucleus. Therefore, St-MIPs were used to replace multi-template molecularly imprinted polymers (Mt-MIPs), making it a simple process with high specificity. Moreover, a method for selective adsorption and determination of lignans in real samples was established by the combination of the obtained St-MIPs as SPE sorbents and HPLC-UV. This study is conducive to a more efficient enrichment of active ingredients in natural products and promotes the industrial application of molecular imprinting techniques for the separation and extraction of natural products.

## 2. Materials and Methods

### 2.1. Materials

*S. chinensis* was purchased from Harbin Medicinal Materials Market (Harbin, China). Standard Schizandrol A, Schisantherin A, Schizandrin A, Schizandrin B, Isoorientin and Isovitexinwere were purchased from Xinyang Zhongjian Metrological Biotechnology Co., Ltd. (Xinyang, China). Choline chloride, iron oxide (II, III) (99.5%), vinyltriethoxysilane, 2-methylpropionitrile, and ethylene glycol dimethacrylate were purchased from Macklin Biochemical Co., Ltd. (Shanghai, China). Ethyl alcohol, and ammonia solution were purchased from Fuyu Fine Chemical Co., Ltd. (Tianjin, China). Tetraethyl orthosilicate (TEOs), acetonitrile, and acetic acid (chromatographic grade) were purchased from Kermel (Tianjin, China). Acrylic acid was purchased from Damao Chemical Reagent Factory (Tianjin, China). Toluene was purchased from Chambroad Petrochemicals Co., Ltd. (Binzhou, China). Acetic acid was purchased from Guangfu Technology Development Co., Ltd. (Tianjin, China). All reagents were of analytical purity grade and were used directly as received without further purification. Methyl alcohol (chromatographic grade) and acetonitrile (chromatographic grade) were purchased from Dikma Co, Ltd. (Beijing, China). Deionized water was purchased from Wahaha Co., Ltd. (Hangzhou, China). Distilled water was produced in-house by the laboratory.

### 2.2. Preparation and Evaluation of Mt-MIPs

#### 2.2.1. Chromatographic Circumstances

The HPLC system (1260-6420 Triple Quad LC/MS, Agilent Technologies, Santa Clara, CA, USA) conditions incorporate a mobile phase of 55% acetonitrile, 45% deionized water and 0.1% acetic acid, a 1.0 mL/min flow, a HiQ sil-C8W (Agilent Technologies, CA, USA), 5 μm, 250 × 4.6 mm analytical column at 25 °C, a 5 μL injection volume and an analysis wavelength of 236 nm. Under the above chromatographic conditions, the separation of column peaks from adjacent peaks was greater than 1.5, and the theoretical tower plate number was not lower than 4000.

#### 2.2.2. Preparation of DES Functional Monomers

Choline chloride (ChCl) was selected as the HBA of the DES, and acrylic acid (AA) was used as the HBD of the DES. ChCl and AA were mixed with a molar ratio of 1:3. The mixture was magnetically stirred at 80 °C until the solution became transparent to obtain the DES ChCl-AA.

#### 2.2.3. Optimization of Polymer Preparation Conditions

In this experiment, the effect of the ratios of template molecules, functional monomers and cross-linkers on the adsorption properties of the polymers were investigated. From the pre-experiment, it was found that the ratio of functional monomer to cross-linkers was 1:5, which gave the largest amount of polymer without wasting the cross-linker. According to Table 1, MIP and NIP were synthesized in different ratios and the synthesized polymers were added to the standard stock solution of template molecules for adsorption.

#### 2.2.4. Synthesis of Mt-MIPs

Fe_3_O_4_ nanoparticles (300 mg) were ultrasonically dispersed in 300.0 mL aqueous ethanol solution (ethyl alcohol/deionized water = 4:1, volume ratio). Then, 4.5 mL of ammonia solution was added quickly, followed by the addition of 3.0 mL of Tetraethyl orthosilicates (TEOs) drop by drop, and mechanical stirring was performed for 6 h in a 50 °C water bath. The reaction was cooled to ambient temperature, the product was separated under the action of an applied magnetic field, washing off the excess silane by ethyl alcohol and distilled water, drying in a vacuum at 40 °C. Then, 300 mg of the products was weighed and dispersed in 30.0 mL toluene, and 3.00 mmol of vinyltriethoxysilane (VTEs) was added, and under nitrogen protection conditions, the reaction was stirred at 110 °C for 12 h. After the end of the reaction, the cooled product was separated under the action of an applied magnetic field, washed and vacuum-dried at 40 °C to obtain vinyl-modified carrier Fe_3_O_4_@SiO_2_@VTEs.

Template molecules (0.25 mmol) and functional monomers (1.23 mmol) were added to 60.0 mL of acetonitrile and pre-assembled at 4 °C for 12 h after sonication for 30 min. Carrier Fe_3_O_4_@SiO_2_@VTEs (0.1 g), cross-linking agent ethylene glycol dimethacrylate (EGDMA, 6,25 mmol) and initiator 2-methylpropionitrile (AIBN, 40 mg) were mixed via sonication for 30 min. Under nitrogen protection, under water bath conditions of 70 °C, mechanical agitation was carried out for 12 h. Under the action of the applied magnetic field, the composite material was separated, the template molecules were eluted by ethyl alcohol/acetic acid (*v*:*v* = 9:1), and the product Mt-MIP was obtained by vacuum-drying at 40 °C.

NIP-1 (blank polymer without template molecules added) was prepared under the same conditions, without the addition of template molecules during preparation.

### 2.3. Preparation and Evaluation of St-MIPs

During the preparation of polymers, the synthetic polymer–specific molecular recognition cavity can selectively rebind with the template molecule or its structural analogues, complementing the template molecule in size, shape, and spatial distribution. The template molecule we selected in this study has a similar parent nucleus, with only a small number of different functional groups. Therefore, in this part of the experiment, one schisandra lignan was chosen as a template, and four structurally similar schisandra lignans were identified at the same time. In addition to this, the synthesis steps of the carrier are optimized in the experiments in this chapter. In the previous experiment, the Fe_3_O_4_ nanoparticles were first wrapped in silica, and then vinyl-modified. This method is the most commonly used method for preparing the core–shell structure of iron tetroxide, but this method is more cumbersome in steps, and the density of the functional groups was relatively low. Therefore, in this part of this study, the one-step method was used to directly wrap the functionalized silica on the outside of the Fe_3_O_4_ nanoparticles and us it as a carrier to synthesize a St-MIP, and then characterize and study the adsorption properties.

#### 2.3.1. Vinyl-Modified Fe_3_O_4_ Was Prepared in One Step

Ethanol (45.0 mL) and deionized water (12.0 mL) were mixed to obtain an aqueous ethanol solution. Fe_3_O_4_ nanoparticles (500 mg) were sonically dispersed in an aqueous ethanol solution, and 7.5 mL of ammonia solution was added quickly, and then stirred well at a speed of 200 r/min. Silicon source (VTEs, 0.01 mol) was diluted with ethanol (45 mL) and added to the above solution drop by drop. Then, the system was stirred continuously for 6 h in a water bath at 45 °C. At the end of the reaction, the reaction system was cooled to ambient temperature. The product was separated under a magnetic field, washed with ethyl alcohol and distilled water to wash off excess silane, and the product Fe_3_O_4_@VSiO_2_ was prepared.

#### 2.3.2. Synthesis of St-MIPs

Template molecules (Schizandrol A, 0.25 mmol) and the functional monomer (ChCl-AA, 1.25 mmol) were added to 60.0 mL of acetonitrile, pre-assembled at 4 °C for 12 h after sonication for 30 min. Carrier Fe_3_O_4_@VSiO_2_ (0.1 g), cross-linker EGDMA (6.25 mmol) and initiator AIBN (40 mg) were sonicated for 30 min. These reacted under nitrogen protection with mechanical agitation in a water bath of 70 °C for 12 h. At the end of the reaction, the system was cooled to an ambient temperature and the composite material was separated under magnetic field. The template molecules were eluded by ethyl alcohol/acetic acid (9:1, *v*:*v*), and after the template molecules were eluded, the polymer was washed to neutral with ethanol, and the product was vacuum-dried at 40 °C to obtain the product St-MIP.

NIP-2 was prepared under the same conditions without template molecules.

### 2.4. Characterization Methods of Mt-MIP and St-MIP

Fourier-transform infrared spectroscopy (FT-IR) was performed on an iS10 (Nicolet, Glendale, WI, USA) instrument to verify the change in functional groups in each stage of polymers synthesis process. Scanning electron microscopy (SEM) was carried out using a Quanta 200 (FEI, Hillsboro, OR, USA) to examine the size and morphology of polymers. Transmission electron microscope (TEM) was used to observe the structure of polymers with a Tecnai G2 S-TWIN (Bruker, Ettlingen, Germany). Thermogravimetric (TG) analysis was conducted by a TGA5500 (TA, New Castle, DE, USA) thermogravimetric analyzer under a nitrogen atmosphere, with the temperature ramping from 30 °C to 800 °C at a rate of 10 °C min^−1^. X-ray powder diffraction (XRD) was used to analyze the change in crystal structure during polymer synthesis by a D/MAX2200 (Rigaku, Tokyo, Japan). The magnetic properties were verified by a vibrating sample magnetometer (VSM).

### 2.5. Adsorption Performance Evaluation of Mt-MIP and St-MIP

The adsorption kinetics of the synthesized Mt-MIP were investigated. The standard solution (Schizandrol A/Schisantherin A/Schizandrin A/Schizandrin B = 1:1:1:1, molar ratio) was prepared with methyl alcohol (chromatographic grade) as the solvent at a concentration of 20 μg/mL. The Mt-MIP and NIP-1 were added to the standard solution of *S. chinensis* lignans, respectively, and adsorbed in a 25 °C water bath for 15 min, 30 min, 45 min, 60 min, 90 min, 120 min, 180 min and 240 min, respectively. The St-MIP and NIP-2 were added to the standard solution of *S. chinensis* lignans, respectively, and adsorbed in a 25 °C water bath for 20 min, 40 min, 60 min, 90 min, 120 min, 180 min and 240 min, respectively. After the polymer was absorbed by the external magnetic field, the clear liquid was absorbed by a syringe, filtered by a 0.22 μm organic filtration membrane, and the peak areas of Schizandrol A, Schisantherin A, Schizandrin A, Schizandrin B were measured by HPLC under the liquid-phase condition described in Section 2.2.1. All tests were repeated three times. The results were substituted into the standard curve to obtain the concentration of standard solution before and after adsorption. And the adsorption quantity *Q* was calculated according to Equation (1).

(1) $$Q=\frac{(C_{0}-C_{1})\times V}{M}$$

*Q* (mg/mL) is the adsorption capacity of *S. chinensis* lignans by the polymer at equilibrium; *C*_0_ (mg/mL) is the initial concentrations of standard solution of *S. chinensis* lignans; *C*_1_ (mg/mL) is the equilibrium concentrations of standard solution of *S. chinensis* lignans. The volume of the adsorbed solution is *V* (mL); the mass of the molecularly imprinted polymer used is *M* (g).

Equilibrium adsorption experiments were performed on the synthesized Mt-MIP and St-MIP. Methyl alcohol (chromatographic grade) was used as the solvent to prepare the standard solution of *S. chinensis* lignans at concentrations of 20 μg/mL, 40 μg/mL, 60 μg/mL, 80 μg/mL and 100 μg/mL (Schizandrol A/Schisantherin A/Schizandrin A/Schizandrin B = 1:1:1:1, molar ratio). Mt-MIP and NIP-1 were added to different concentrations of *S. chinensis* lignans standard solution and shaken in a water bath at 25 °C for 90 min. St-MIP and NIP-2 were added to different concentrations of *S. chinensis* lignans standard solution and shaken in a water bath at 25 °C for 120 min. The peak areas of the four *S. chinensis* lignans were obtained by measuring the filtered clear solution using HPLC. And the adsorption capacities *Q* were calculated according to Equation (1).

Selective adsorption experiments were performed on the synthesized Mt-MIP. Isoorientin and Isovitexin were present in the *S. chinensis* fruits extracts in addition to the four lignans. In this study, we hoped that the MIP using lignans as templates would be able to efficiently separate the four lignans from the *S. chinensis* fruits extracts. Therefore, Isoorientin and Isovitexin were used as selective adsorption experiments to determine the effect of interference. A certain concentration of mixed standard solution of *S. chinensis* lignans with Isoorientin and Isovitexin was prepared (Schizandrol A/Schisantherin A/Schizandrin A/Schizandrin B/Isoorientin/Isovitexin = 1:1:1:1:1:1, molar ratio). Mt-MIP and NIP-1 were added to the standard solution, respectively, and adsorption was performed by shaking it in a water bath at 25 °C for 90 min. St-MIP and NIP-2 were added to the standard solution, respectively, and adsorption was performed by shaking it in a water bath at 25 °C for 120 min. The peak areas of the four *S. chinensis* lignans, Isoorientin and Isovitexin were obtained by measuring the filtered clear solution using HPLC. And the adsorption capacities were calculated according to Equation (1).

### 2.6. S. chinensis Lignans Separation by St-MIP Combined with SPE

Magnetic SPE, as a novel SPE separation method, has received more and more attention in the fields of natural products separation. St-MIPs can use only one template to separate multiple natural products at the same time, which is convenient and cost-effective. Therefore, in this study, the St-MIP synthesized with Schizandrol A as the template molecule was selected as the filler in SPE.

St-MIPs (200 mg) were added to a Methyl alcohol/water (80:20, *v:v*, 2.0 mL) loading solvent; after the water bath oscillated for 90 min, the lower end of the extraction column with a diameter of 0.5 cm was filled with cotton and filter paper as support, and the upper end was filled with cotton to prevent the filler from floating. After the loading solvent flowed out, the loaded small column was washed with water as an eluent at a flow rate of 1.0 mL/min. After the eluent flowed out, the loaded SPE column was eluted with 2.0 mL an ethyl alcohol/acetic acid (85:15, *v:v*) solution at a flow rate of 0.5 mL/min. A solid-phase extraction column filled with a St-MIP was obtained.

## 3. Results

### 3.1. Standard Curves of S. chinensis Lignans

The standard solution of 1.00 mg/mL was obtained by accurately weighing 10 mg of Schizandrol A (99.2%, Xinyang Zhongjian Metrological Biotechnology Co., Ltd., Lot#:200517), dissolving it with methyl alcohol (Chromatographic grade), and fixing the volume. The standard stock solution of 1.00 mg/mL was diluted to obtain a series of standard solutions of different concentrations, and then filtered through a 0.22 μm organic system filter membrane and loaded into liquid-phase vials and refrigerated. The concentrations of the standard stock solution were determined according to the liquid-phase conditions in Section 2.2.1, and linear regression was performed with the concentration of Schizandrol A as the horizontal coordinate and the peak area as the vertical coordinate to obtain the standard curve of Schizandrol A, as shown in Table 2.

The standard curves of Schisantherin A, Schizandrin A, Schizandrin B were prepared in the same way as above, and the obtained standard curves are also shown in Table 2.

### 3.2. Results of Optimization of Polymer Preparation Conditions

**Figure 1:**
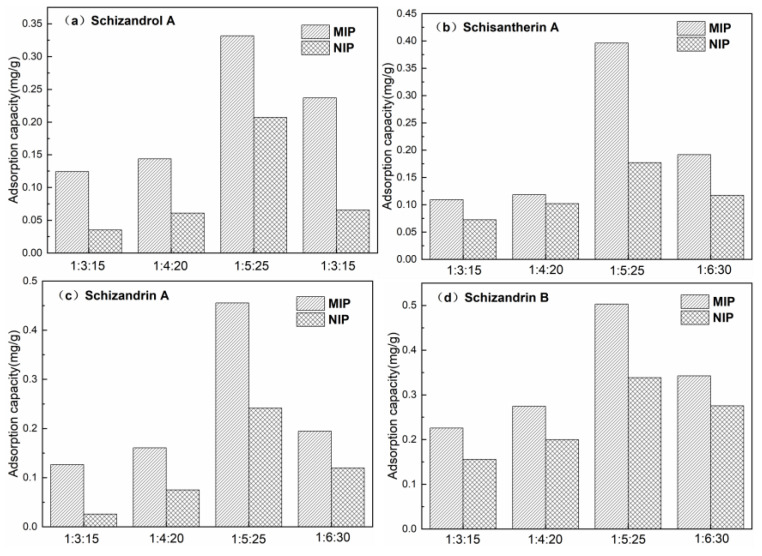
Adsorption capacity of polymers in different proportions: Schizandrol A (**a**); Schisantherin A (**b**); Schizandrin A (**c**); Schizandrin B (**d**).

The effects of different ratios of template molecules (Schizandrol A/Schisantherin A/Schizandrin A/Schizandrin B = 1:1:1:1, molar ratio), functional monomers and cross-linkers on the adsorption properties of the synthesized polymers are shown in Figure 1.

At a fixed amount of template molecule, the adsorption capacity of the polymer on the template molecule gradually increased with the increase in the amount of functional monomer ChCl-AA and cross-linker EGDMA, reaching the highest capacity at the ratio of 1:5:25, and then the adsorption capacity of the polymer gradually became smaller after the ratio was increased to 1:6:30. When the amount of ChCl-AA was small, the amount of complex formed with the template molecule was small, which resulted in fewer adsorption sites after the formation of the polymer, leading to a low adsorption capacity. When the amount of ChCl-AA was gradually increased, the template molecule complexed with ChCl-AA more fully, forming more adsorption sites, and the adsorption amount of the polymer also increased. However, when the amount of functional monomer was too much, the amount of functional monomer themselves increased, which reduced the specific sites of the polymers, thus affecting the adsorption capacity of the polymers. When the amount of cross-linker EGDMA was low, the mechanical properties of the synthesized polymers were poor, and at the same time, the low amount of cross-linker led to fewer adsorption sites, which made the adsorption properties of the polymers worse. However, the addition of too much cross-linkers may lead to excessive cross-linking of the polymer, which also affects the adsorption properties of the polymer. Therefore, the optimal ratio of 1:5:25 was chosen as the best ratio for polymer synthesis.

### 3.3. Template Molecular Screening of St-MIPs

In the preparation process of St-MIPs, the adsorption properties of the polymers synthesized with Schizandrol A, Schisantherin A, Schizandrin A and Schizandrin B as template molecules were studied, and the adsorption results are shown in Figure S1. The polymer prepared with single *S. chinensis* lignans as the template has the ability to recognize Schizandrol A, Schisantherin A, Schizandrin A and Schizandrin B. When Schizandrol A, Schisantherin A, and Schizandrin B were used as the template molecule, the recognition ability of Schizandrin A was the strongest. When Schizandrin A was used as the template, the recognition ability of Schizandrin B was the strongest. From the perspective of the overall adsorption capacity, when choosing Schizandrol A as the template molecule, the total amount of the four kinds of *S. chinensis* lignans’ adsorption was highest. Therefore, in the following experiment, Schizandrol A was used as the template molecule to prepare St-MIPs to replace Mt-MIPs and adsorb four kinds of *S. chinensis* lignans in the solution at the same time.

### 3.4. Characterization of Mt-MIP and St-MIP

#### 3.4.1. FT-IR Spectroscopy Analysis

Fourier-transform infrared spectroscopy was used to characterize the Fe_3_O_4_, Fe_3_O_4_@SiO_2_, Fe_3_O_4_@SiO_2_@VTEs, Mt-MIP, NIP-1 Fe_3_O_4_@VSiO_2_, St-MIP, NIP-2 in order to validate the functional group modifications throughout the synthesis of Mt-MIP and St-MIP.

**Figure 2:**
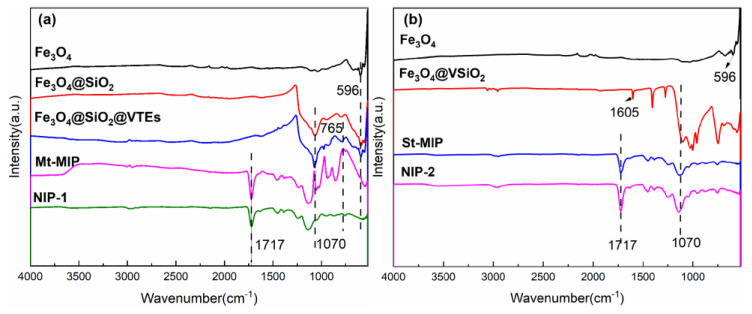
FT-IR spectra of Mt-MIP (**a**) and St-MIP (**b**).

The results are shown in Figure 2. The characteristic absorption peak of Fe-O appeared at 596 cm^−1^ and was present in the subsequent silica modification as well as in the synthesized polymers, indicating that the Fe_3_O_4_ nanoparticles were very stable during the synthesis of MIP and did not change due to the change in external conditions. The Fe_3_O_4_@SiO_2_ in Figure 2a shows a clear anti-symmetric stretching vibration peak of Si-O-Si at 1070 cm^−1^, indicating that SiO_2_ was successfully wrapped outside Fe_3_O_4_ by the hydrolysis of tetraethyl orthosilicate. And the characteristic peak of SiO_2_ remained during the synthesis of the polymer afterwards, indicating that SiO_2_ was not affected during the polymerization process. After vinyl-modification of Fe_3_O_4_@SiO_2_, Fe_3_O_4_@SiO_2_@VTEs were obtained, and the characteristic peak of C=C at 765 cm^−1^ could be observed. However, in Figure 2b, in Fe_3_O_4_@VSiO_2_, not only does the anti-symmetric stretching vibration peak of Si-O-Si appear at 1070 cm^−1^, but the characteristic peak of C=C at 1605 cm^−1^ is also shown, which indicates the successful use of one-step method to successfully wrap the functionalized SiO_2_ around the exterior of Fe_3_O_4_. The FT-IR spectra of the synthesized MIP and NIP are essentially the same, which is due to the fact that no template molecules were added to the NIP, while the MIP, after eluting of the template, also does not contain template molecules. The presence of the C=O absorption peak in EGDMA at 1717 cm^−1^, combined with the presence of Fe and Si characteristic peaks, also indicates the successful synthesis of Mt-MIP and St-MIP, and the simplicity of the one-step method of wrapping functionalized SiO_2_ directly on the exterior of Fe_3_O_4_ nanoparticles as a carrier.

#### 3.4.2. Surface Morphology Analysis of Mt-MIP and St-MIP

**Figure 3:**
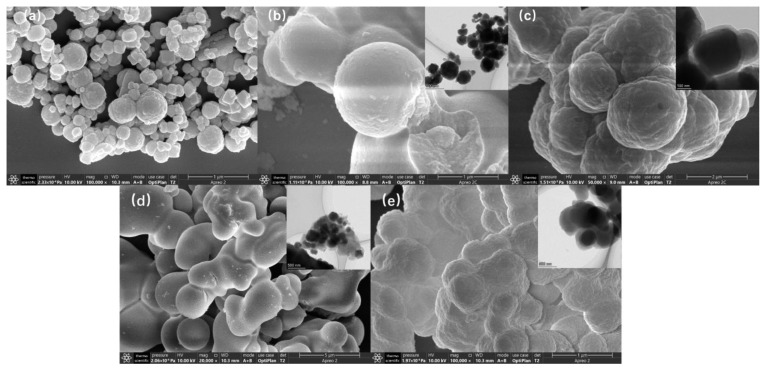
Surface morphology analysis of Fe_3_O_4_ (**a**), Fe_3_O_4_@SiO_2_ (**b**), Mt-MIP (**c**), Fe_3_O_4_@VSiO_2_ (**d**), and St-MIP (**e**). PS: The TEM image of the material is in the upper right corner of the SEM image of the corresponding material.

The morphology of Mt-MIP and St-MIP was characterized by transmission electron microscopy and scanning electron microscopy, and the results are shown in Figure 3. Figure 3a shows the SEM of Fe_3_O_4_. It can be seen that although the magnetic Fe_3_O_4_ nanoparticles have some agglomeration, the nanoparticles are generally clear and the diameter is essentially not higher than 500 nm. Figure 3b shows the Fe_3_O_4_ nanoparticles coated with silica. It can be clearly seen that the particle surface was obviously smooth compared with that of Fe_3_O_4_, and the particle diameter was significantly increased. Figure 3c shows the scanning electron microscopy of Mt-MIP. The diameter can reach more than 1 μm, which was significantly increased compared with the previous one. As can be seen from the transmission electron microscopy of Mt-MIP, the thickness of the outermost inclusion increases significantly, which also further increases the particle size. The morphology of Mt-MIP synthesized from the carrier raw material Fe_3_O_4_ to the final Mt-MIP was spherical, which also indicates that each step of the reaction was carried out on the surface of the magnetic carrier; so, the morphology did not change. In Figure 3d, it can be seen that the external surface of Fe_3_O_4_@VSiO_2_ was relatively smooth, and it can be further observed that the Fe_3_O_4_ cores were encapsulated in the inserted TEM image. After a series of operations, the surface of St-MIP in Figure 3e was significantly rougher than that of Fe_3_O_4_@VSiO_2_, and it could be seen from the TEM image that the outer envelope of St-MIP was significantly thickened, and two translucent layers appeared on the outside of Fe_3_O_4_ core. The results show that St-MIP was successfully coated on silica by the one-step method.

#### 3.4.3. TG and DTG Analysis of Mt-MIP and St-MIP

**Figure 4:**
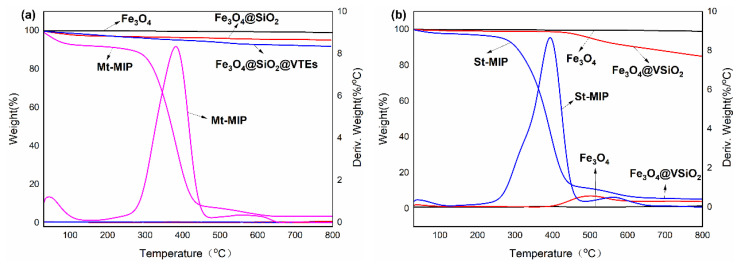
TG and DTG analysis of Mt-MIP (**a**) and St-MIP (**b**).

The structure and the thermal stability of Mt-MIP and St-MIP were further analyzed in this study using thermogravimetric analysis, and the results are shown in Figure 4. Under the protection of N_2,_ the weight loss of Mt-MIP and St-MIP from ambient temperature to 800 °C was analyzed, respectively. Fe_3_O_4_ only exhibited a small part of the weight loss corresponding to the bounded water and impurities in nanoparticles during the whole heating process. The melting point of SiO_2_ is above 1000 °C; so, Fe_3_O_4_@SiO_2_ and Fe_3_O_4_@SiO_2_@VTEs only demonstrate part of the weight loss owing to bounded water and impurities. The weight loss of Fe_3_O_4_@ weight loss was 0.678% when the temperature reached 110 °C, mainly due to the evaporation of water. As the temperature increased, the weight loss rate was fastest at 400–800 °C, with a weight loss of 13.657%. At this stage, the weight loss was mainly caused by vinyl, which also indicates that the SiO_2_-Fe_3_O_4_ core–shell structure with vinyl was successfully prepared by the one-step method. The weight loss of Mt-MIP was 7.139% at 110 °C, while that of St-MIP was 2.312% at 110 °C, which was mainly due to the evaporation of water from the polymer. At 250–700 °C, both Mt-MIP and St-MIP started to lose weight rapidly, mainly because the DES and EGDMA contained in MIP decomposed at high temperature. And the remaining part of the mass was the carrier wrapped in the polymer, which proved that the synthesized polymer was successfully wrapped on the carrier, and the MIP could withstand a temperature below 200 °C with good thermal stability. Therefore, the polymers exhibit relatively high thermal stability.

#### 3.4.4. VSM Analysis of Mt-MIP and St-MIP

**Figure 5:**
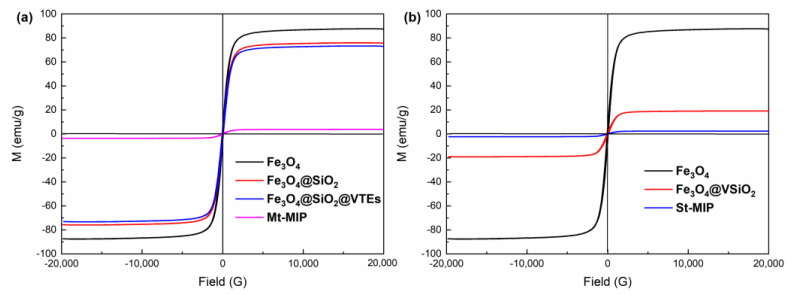
Hysteresis loops for each stage of Mt-MIP (**a**) and St-MIP (**b**).

To study the magnetic properties of Mt-MIP and St-MIP, VSM was used in measuring the hysteresis loop of the samples. Magnetic Fe_3_O_4_ was used as a carrier to synthesize molecularly imprinted polymers for adsorption separation, in which magnetism was the most obvious advantage over other carriers, which can be separated quickly by an applied magnetic field, making the separation more rapid and convenient. It can be seen from Figure 5 that the magnetic intensity of the samples increases with the increase in the magnetic field. The saturation magnetic intensities of Mt-MIP and St-MIP are 3.7391 emu/g and 2.3742 emu/g, respectively. Compared with Fe_3_O_4_ nanoparticles, Fe_3_O_4_@SiO_2_ and Fe_3_O_4_@SiO_2_@VTEs, Fe_3_O_4_@VSiO_2_, Mt-MIP and St-MIP showed a decreasing trend of saturation magnetic intensity with each modification. This was attributed to the fact that with each modification, a non-magnetic layer was wrapped around the Fe_3_O_4_ nanoparticles, which reduces the magnetic properties compared with Fe_3_O_4_. However, it can be seen from the figure that the magnetic curves of all the materials pass from the origin and are symmetrical without any obvious hysteresis. This indicates that the materials synthesized are superparamagnetic, and in combination with Figure S2, showing the XRD analysis of Mt-MIP and St-MIP, it can be demonstrated that the synthesized MIPs are still magnetic and can be separated quickly in the experiment using an applied magnetic field.

### 3.5. Adsorption Capacity Evaluation of Mt-MIP and St-MIP

**Figure 6:**
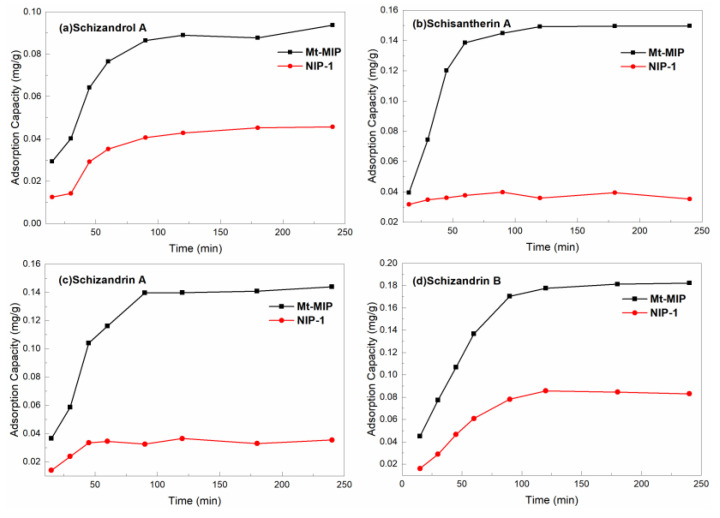
Adsorption kinetic curves of Mt-MIP and NIP-1.

The adsorption kinetic curves of Mt-MIP and NIP-1 on *S. chinensis* lignans standards are shown in Figure 6. It is obvious that the adsorption capacity of four S. chinensis lignans over Mt-MIP increased gradually and reached saturation within 90–120 min. Considering the trend of adsorption effects, 90 min was chosen as the optimal adsorption time. The imprinted polymer was uneven when wrapping around the surface of the carrier, leading to different depths of internal cavities. Therefore, during the adsorption process, the external imprinted cavities first contacted the pentosidine and performed rapid adsorption. The external cavities were gradually filled with time, and the internal cavities needed more time for mass transfer; so, the efficiency decreased and reached equilibrium after 90–120 min. The adsorption trend of NIP-1 was the same as Mt-MIP, but the apparent adsorption capacity was significantly lower than that of NIP-1. This is due to the fact that NIP-1 did not have specific adsorption capability, mainly relying on the polymer surface for a small capacity of adsorption.

The relationship between the apparent equilibrium adsorption capacity and adsorption time of the molecularly imprinted polymer for the adsorption of *S. chinensis* lignans was investigated using adsorption kinetics. Quasi-primary kinetic equations and quasi-secondary kinetic equations were fitted, respectively. The model equations were as follows:

(2) $$\mathrm{Quasi-primary}\;\mathrm{kinetic}\;\mathrm{quations}:\ln\left(Q_{e}-Q_{t}\right)=\ln Q_{e}-k_{1}t$$

(3) $$\mathrm{Quasi-secondary}\;\mathrm{kinetic}\;\mathrm{equations}:\;\frac{t}{Q_{t}}=\frac{1}{k_{2}Q_{e}^{2}}+\frac{e}{Q_{e}}$$

*Qe* (mg/g) is the equilibrium adsorption capacity; *Q_t_* (mg/g) is the adsorption capacity for adsorption time t; *k*_1_ (1/min) is the rate constant of first-order kinetics; *t* (min) is the adsorption time; *k*_2_ (g/(mg·min)) is the rate constant of second-order.

Table S1 and Figure S3 show that the *R*^2^ values of the equations fitted using quasi-secondary kinetics are all greater than 0.90, significantly higher than those fitted using quasi-first-order kinetics. The adsorption capacity fitted using the quasi-secondary kinetic model was closest to the actual data. Therefore, the quasi-secondary kinetic equation was the most suitable for the simulation of the adsorption kinetics of Mt-MIP.

**Figure 7:**
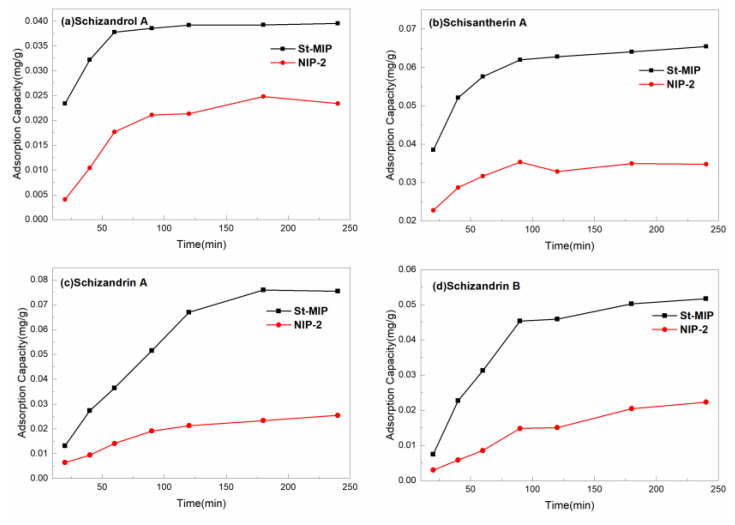
Adsorption kinetic curves of St-MIP and NIP-2.

The adsorption kinetic curves of St-MIP and NIP-2 on *S. chinensis* lignans standard solution are shown in Figure 7. The adsorption of Schizandrol A, Schisantherin A, Schizandrin B by St-MIP increased at the initial 90 min, and reached the adsorption equilibrium at about 90 min. After 120 min, the adsorption of the four *S. chinensis* lignans over St-MIP essentially ceased to increase. The adsorption over NIP-2 reached equilibrium at 120 min. The adsorption capacity of St-MIP was larger than that of NIP-2 during the whole adsorption process, which indicated that there was no specific adsorption site in NIP-2. In order to further investigate the adsorption behavior of *S. chinensis* lignans over St-MIP, the quasi-primary kinetic model and quasi-secondary kinetic model were used to further simulate the adsorption dose–time curves of St-MIP. From the fitted results in Table S2 and Figure S4, it was observed that the *R^2^* of the quasi-secondary kinetic equation was closer to 1; it is therefore concluded that the adsorption process of St-MIP belonged to the adsorption kinetic secondary reaction.

**Figure 8:**
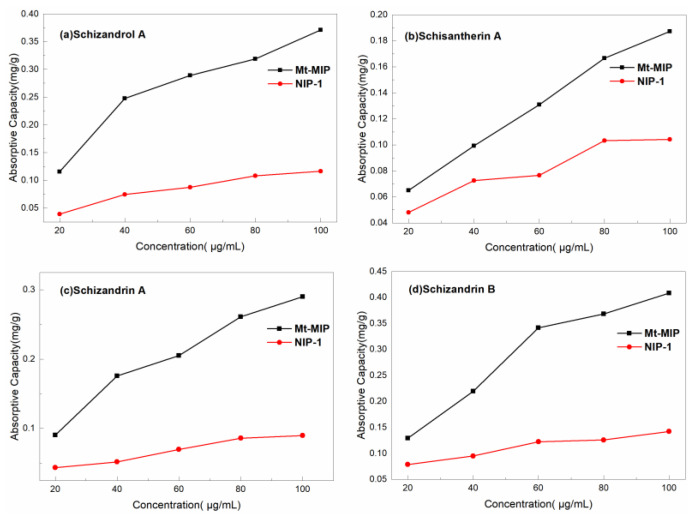
Adsorption isothermal curves of Mt-MIP and NIP-2.

Figure 8 shows the adsorption isothermal curves of *S. chinensis* lignans over Mt-MIP and NIP-2 with the concentration of the standard solution in range of 20 μg/mL–100 μg/mL. It can be seen that the adsorption capacity of Mt-MIP increased with the increased concentration of *S. chinensis* lignans. At the concentration of 100 μg/mL, the adsorption capacity of Schizandrol A, Schisantherin A, Schizandrin A, and Schizandrin B was 0.371 mg/g, 0.1874 mg/g, 0.2901 mg/g and 0.1423 mg/g, respectively. However, the apparent adsorption capacity of NIP-2 was significantly lower than that of Mt-MIP, indicating that Mt-MIP possessed good selectivity.

In the process of adsorption, the relationship between the solution concentration *C_e_* and the adsorption capacity *Q_e_* can be expressed by the adsorption isotherms. The common adsorption isotherms include the Langmuir model and Freundlich model with the following equations:

(4) $$\mathrm{Langmuir}\;\mathrm{model}:\;\frac{C_{e}}{Q_{e}}=\frac{C_{e}}{Q_{max}}+\frac{1}{Q_{max}k_{L}}$$

(5) $$\mathrm{Freundlich}\;\mathrm{model}:\;\ln Q_{e}=\ln k_{F}+\frac{1}{n}\ln C_{e}$$

*Q_max_* (mg/g) is the maximum adsorption of the molecularly imprinted polymer; *k_L_* (mL/mg) is the equilibrium adsorption constant of the Langmuir model; *k_F_* and n are both empirical constants of adsorption of the Freundlich model.

The Langmuir model (4) and Freundlich model (5) were used to fit the above results, shown in Table S3 and Figure S5. It can be seen that the correlation coefficient *R^2^* of the Freundlich model’s fitting results of adsorption isotherms for Mt-MIP and NIP-1 was higher than that of the Langmuir model by comparing the data in the table. The correlation coefficients of the fitting results are all above 0.9, indicating a multilayer adsorption process. The adsorption sites on the polymer surface are not uniform. For *S. chinensis* lignans, there are binding sites with different affinities in the imprinted cavity, and Schizandrol A, Schisantherin A, Schizandrin A, and Schizandrin B have competitive adsorption relationship in the solution.

**Figure 9:**
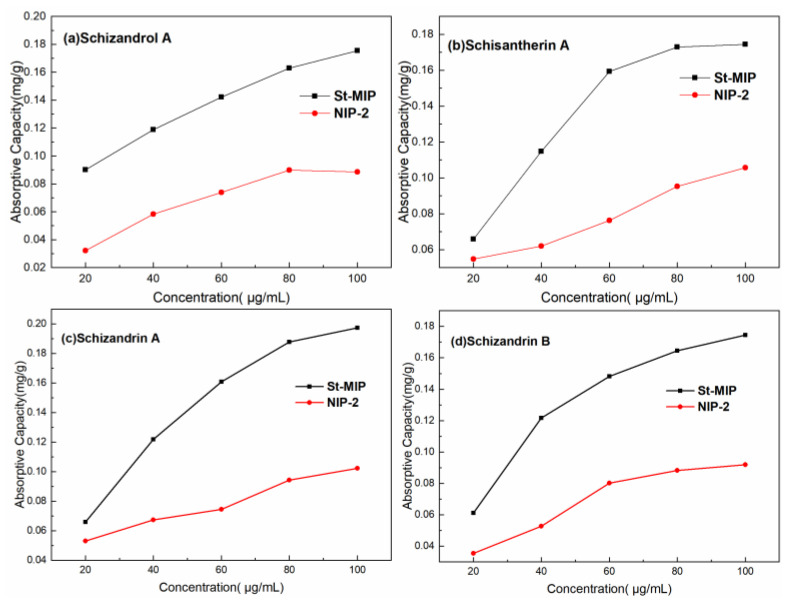
Adsorption isothermal curves of St-MIP and NIP-2.

The adsorption curves over St-MIP are shown in Figure 9. In this experiment, the changes in the adsorption capacity of St-MIP on the four types of *S. chinensis* lignans were investigated between 20 μg/mL and 100 μg/mL. The adsorption capacity of St-MIP for Schizandrol A, Schisantherin A, Schizandrin A, and Schizandrin B increased with the increase in the concentration of standard solution, and finally reached the adsorption equilibrium at 100 μg/mL, which was 0.17534 mg/g, 0.17434 mg/g, 0.1972 mg/g and 0.17445 mg/g, respectively. The adsorption capacity of NIP-2 generally increased with increasing concentration, but the apparent adsorption capacity was also lower than that of St-MIP, indicating that the imprinted cavities in St-MIP can recognize substances with a similar molecular structure to the template and have good adsorption performance. Table S4 and Figure S6 show the fitting results of the Langmuir model (4) and the Freundlich model (5) for the isothermal adsorption curve of St-MIP. It can be seen from the table that the correlation coefficients fitted by the Freundlich model are higher compared with those of the Langmuir model. It is shown that the Freundlich model (5) was more suitable to describe the adsorption process over St-MIP. The adsorption of Schizandrol A, Schisantherin A, Schizandrin A, and Schizandrin B was a non-homogeneous multilayer adsorption, during which the four *S. chinensis* lignans demonstrated competitive adsorption.

### 3.6. Selective Adsorption over Mt-MIP and St-MIP

In the selective adsorption experiments, Isovitexin and Isoorientin were selected as the reference materials for competitive adsorption, and the structures of both are shown in Figure S7.

The results of selective adsorption of Mt-MIP and NIP-1 are shown in Figure S8a. It can be seen that Mt-MIP exhibited the highest capacity of *S. chinensis* lignans in the standard mixture, which was significantly higher than that of NIP-1. The total adsorption capacities of Mt-MIP and NIP-1 for Isovitexin and Isoorientin were similar without much difference. These results indicate that MIP exhibits recognition ability for template molecules and greatly exceeds NIP-1.

In order to further study the specificity of St-MIP for the adsorption of *S. chinensis* lignans, selective adsorption of *S. chinensis* lignans, Isoorientin and Isovitexin was carried out, as shown in Figure S8b. St-MIP in the mixed solution of *S. chinensis* lignans, Isovitexin and Isoorientin still had a high adsorption capacity of *S. chinensis* lignans. The adsorption capacity of Isovitexin and Isoorientin was similar between St-MIP and NIP-2. This indicates that St-MIP prepared using a single template, Schisandrin A, still maintains specific adsorption for Schisantherin A, Schizandrin A, and Schizandrin B with a similar parent nucleus.

### 3.7. Re-Usability and Stability Evaluation of Mt-MIP and St-MIP

**Figure 10:**
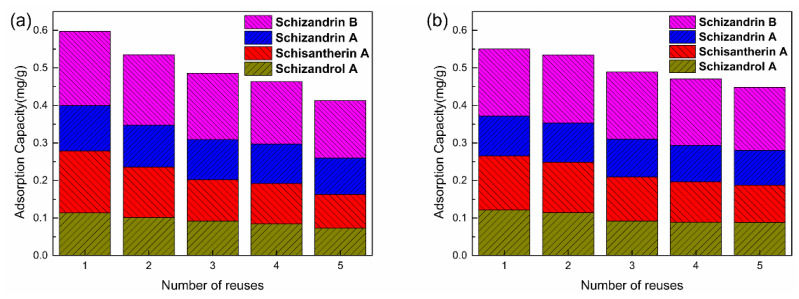
Adsorption properties of recycle Mt-MIP (**a**) and St-MIP (**b**).

The stability and re-usability of the Mt-MIP and St-MIP are crucial for their subsequent applications. Therefore, in this experiment, 20 mg of Mt-MIP and St-MIP was added into 20 μg/mL of *S. chinensis* lignans’ standard solution, and shaken in a water bath at 25 °C for 90 min. The adsorbed *S. chinensis* lignans in Mt-MIP and St-MIP were further eluted and the adsorption–elution experiments were performed five times. After the five repeated tests, the adsorption amount of Mt-MIP and St-MIP remained 69.78% and 81.52% of the initial adsorption amounts, respectively. As shown in Figure 10, as the adsorption–elution tests were repeated, the adsorption capacities for all four *S. chinensis* lignans decreased. This may be due to the fact that the MIP will be lost to some extent during the repeated adsorption–elution process, and there will also be a part of the template in the blotting cavity that is not washed out after adsorption. In general, MIP was stable and can be re-used in the experiment.

### 3.8. Solid-Phase Extraction Results

**Figure 11:**
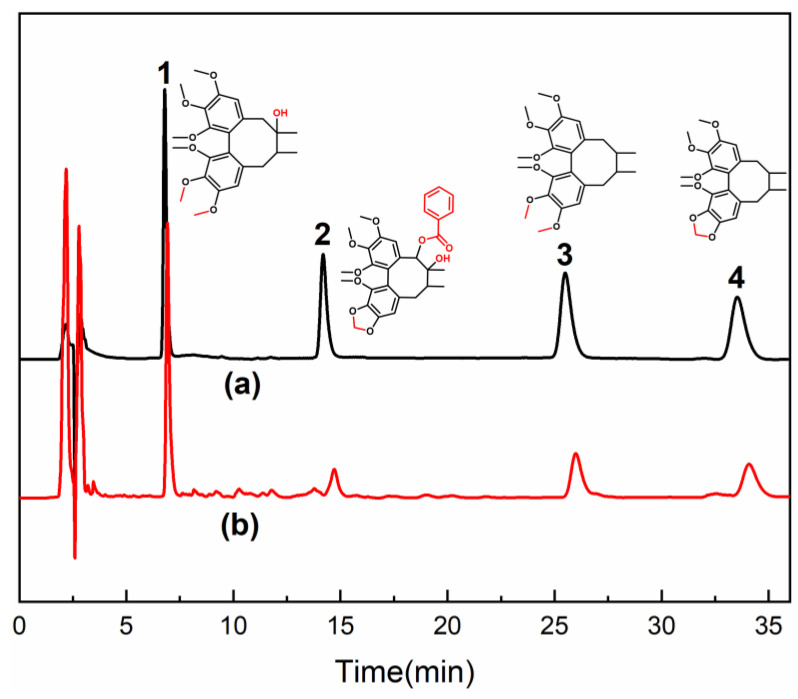
HPLC of lignan standards (a) and *S. chinensis* sample after St-MIP SPE (b): 1—Schizandrol A, 2—Schisantherin A, 3—Schizandrin A, 4—Schizandrin B.

Schizandrol A, Schisantherin A, Schizandrin A, and Schizandrin B in *S. chinensis* extracts were adsorbed by the St-MIP column. And the St-MIP column was eluted by an ethyl alcohol/acetic acid (85:15, *v:v*) solution. The eluent was determined by HPLC, as shown in Figure 11. By substituting the contents of *S. chinensis* lignans in the eluent into the standard curve equation of 3.1, the contents of Schizandrol A, Schisantherin A, Schizandrin A, and Schizandrin B were 0.1335 mg/mL, 0.03474 mg/mL, 0.0653 mg/mL and 0.06267 mg/mL, respectively.

The purity of four kinds of *S. chinensis* lignans after St-MIP SPE was calculated by Equation (6). The purity of the *S. chinensis* total lignans after SPE was 74.05%. The purity of Schizandrol A, Schisantherin A, Schizandrin A, and Schizandrin B was 33.38%, 8.69%, 16.33% and 15.67%, respectively.

(6) $$P=\frac{C_{H}\times V}{m_{0}}$$

*P* (%) is the purity of *S. chinensis* lignans; *C_H_* (mg/mL) is the concentration of *S. chinensis* lignans by HPLC; *V* (mL) is the volume of the extraction solution; *m*_0_ (mg) is the total mass of 1.0 mL of extraction solution after drying.

## 4. Conclusions

In this study, firstly, Mt-MIP was synthesized using Schizandrol A, Schisantherin A, Schizandrin A, and Schizandrin B as templates, ChCl-AA as functional monomers, and the core–shell-structured polymer formed by a vinyl–silicon source modified by magnetic Fe_3_O_4_ as a carrier. Based on the structural characteristics of the *S. chinensis* lignans, one kind of lignan compound was used as a single template, and the one-step method preparation of magnetic St-MIP was carried out. Then, the characterization of magnetic Mt-MIP illustrated that the synthesized Mt-MIP has high adsorption capacity, stability, and selectivity for the four *S. chinensis* lignans. While the characterization of magnetic St-MIP illustrated that the St-MIP with Schizandrol A as the template could still adsorb four *S. chinensis* lignans with a similar parent nucleus, which not only had high adsorption capacity, stability and selectivity, but also saved costs. The total recovery of *S*. *chinensis* lignans extracted by this method was 96.78%. The recoveries of Schizandrol A, Schisantherin A, Schizandrin A, and Schizandrin B were 94.26%, 93.13%, 98.75% and 98.55%, respectively. The purity of the total *S. chinensis* lignans after SPE was 74.05%. Among them, the purity of Schizandrol A, Schisantherin A, Schizandrin A, and Schizandrin B was 33.38%, 8.69%, 16.33% and 15.67%, respectively. Finally, St-MIP was combined with SPE for Schizandrol A, Schisantherin A, Schizandrin A, and Schizandrin B separation from *S. chinensis* lignans’ extracts.

This study provides a theoretical basis for multi-lignans compounds’ separation with magnetic St-MIP from *S. chinensis* extracts. It also developed a novel idea for the purification of natural products with a similar parent nucleus by MIT. Further research should focus on finding cheaper templates, exploring simpler preparation methods to reduce experimental costs. The template leakage problem also needs to be solved.

## Figures and Tables

**Table 1:** Molecularly imprinted polymers prepared under different polymerization conditions.

Polymers	Molar Ratio ^a^	Functional Monomers	Cross-Linkers
MIP1/NIP1	1:3:15	ChCl-AA	EDGMA
MIP2/NIP2	1:4:20	ChCl-AA	EDGMA
MIP3/NIP3	1:5:25	ChCl-AA	EDGMA
MIP4/NIP4	1:6:30	ChCl-AA	EDGMA

Ps: ^a^ molar ratio: template molecules: functional monomers: cross-linkers.

**Table 2:** Standard curves of *S. chinensis* lignans.

Name	Standard Curve Equation	*R* ^2^	Linear Detection Range (μg/mL)	Retention Time (min)
Schizandrol A	*Y* = −86.814 + 2.5790 × 10^4^*X*	0.9999	7.81~1000	6.7
Schisantherin A	*Y* = −145.5 + 2.4182 × 10^4^*X*	0.9999	7.81~1000	13.5
Schizandrin A	*Y* = −25.482 + 2.9300 × 10^4^*X*	0.9999	7.81~1000	23.0
Schizandrin B	*Y* = −153.98 + 2.7309 × 10^4^*X*	0.9999	7.81~1000	31.0

## Data Availability

The data supporting the findings in this study are contained within this article or in the Supplementary Materials.

## Supplementary Materials

The following supporting information can be downloaded at: https://www.mdpi.com/article/10.3390/polym16223124/s1, Figure S1: Template molecular screening of St-MIPs; Figure S2: XRD analysis of Mt-MIP (a) and St-MIP (b); Figure S3: Kinetic fitting curves for Mt-MIP and NIP-1: (a) first-order kinetic fitting curves; (b) second-order kinetic fitting curves; Figure S4: Kinetic fitting curves for St-MIP and NIP-2: (a) first-order kinetic fitting curves; (b) second-order kinetic fitting curves; Figure S5: Adsorption isotherm fitting curves for Mt-MIP and NIP-1: (a) Langmuir model fitting curves; (b) Freundlich model fitting curves; Figure S6: Adsorption isotherm fitting curves for St-MIP and NIP-2: (a) Langmuir model fitting curves; (b) Freundlich model fitting curves; Figure S7: Chemical structure of Isovitexin (a) and Isoorientin (b); Figure S8: Selective adsorption of MIPs: adsorption capacities of *S. chinensis* lignans and flavonoids by Mt-MIP and NIP-1 (a); adsorption capacities of *S. chinensis* lignans and flavonoids by St-MIP and NIP-2 (b); Table S1: Kinetic fitting parameters of Mt-MIP and NIP-1; Table S2: Kinetic fitting parameters of St-MIP and NIP-2; Table S3: Adsorption isothermal model parameters of Mt-MIP and NIP-1; Table S4: Adsorption isothermal model parameters of St-MIP and NIP-2.
