# Supplementary material for: Magnetic Molecularly Imprinted Polymer Combined with Solid-Phase Extraction for Purification of Schisandra chinensis Lignans
Source: Polymers (Basel). 2024 Nov 8;16(22):3124. doi: 10.3390/polym16223124 (PMC11598105; doi:10.3390/polym16223124)
Supplement: Supplementary file 1 [file polymers-16-03124-s001.zip › polymers-3249839-supplementary.pdf]

Magnetic Molecularly Imprinted Polymer Combined with Solid-Phase Extraction for Purification of *Schisandra chinensis* Lignans

Huijuan Xu [1,†], Lihan Sun [1,†], Yufei Du [1], Wenxin Duan [1], Wei Li [1], Sha Luo [1], Bing Liang [2], Chunhui Ma [1,*] and Gaofeng Pan [2,*]

[1] Key Laboratory of Bio-Based Material Science and Technology, Ministry of Education, College of Material Science and Engineering, Northeast Forestry University, Harbin 150040, China; xuhj0323@163.com (H.X.); sunlihan@163.com (L.S.); duyufei@163.com (Y.D.); duanwenxin@163.com (W.D.); liwei19820927@126.com (W.L.); luo.sha.85@163.com (S.L.)

[2] Mudanjiang Heng Feng Paper Co., Ltd., Mudanjiang 157013, China; liangbing@163.com

* Correspondence: machunhui@nedu.edu.cn (C.M.); hengfengpaper6971@163.com (G.P.); Tel./Fax: +86-451-82191204 (C.M.); +86-453-6886771 (G.P.)

† These authors contributed equally to this work.

Catalog

Figure S1 Template molecular screening of St-MIPs 2

Figure S2 XRD analysis of Mt-MIP (a) and St-MIP (b) 3

Figure S3 Kinetic fitting curves for Mt-MIP and NIP-1: (a) First-order kinetic fitting curves; (b) Second-order kinetic fitting curves. 4

Figure S4 Kinetic fitting curves for St-MIP and NIP-2: (a) First-order kinetic fitting curves; (b) Second-order kinetic fitting curves. 5

Figure S5 Adsorption isotherm fitting curves for Mt-MIP and NIP-1: (a) Langmuir model fitting curves; (b) Freundlich model fitting curves. 6

Figure S6 Adsorption isotherm fitting curves for St-MIP and NIP-2: (a) Langmuir model fitting curves; (b) Freundlich model fitting curves. 7

Figure S7 Chemical structure of Isovitexin (a) and Isoorientin (b) 8

Figure S8 Selective adsorption of MIPs: adsorption capacities of *S. chinensis* lignans and flavonoids by Mt-MIP and NIP-1 (a); adsorption capacities of *S. chinensis* lignans and flavonoids by St-MIP and NIP-2 (b) 9

Table S1: Kinetic fitting parameters of Mt-MIP and NIP-1 10

Table S2: Kinetic fitting parameters of St-MIP and NIP-2 11

Table S3: Adsorption isothermal model parameters of Mt-MIP and NIP-1 12

Table S4: Adsorption isothermal model parameters of St-MIP and NIP-2 13

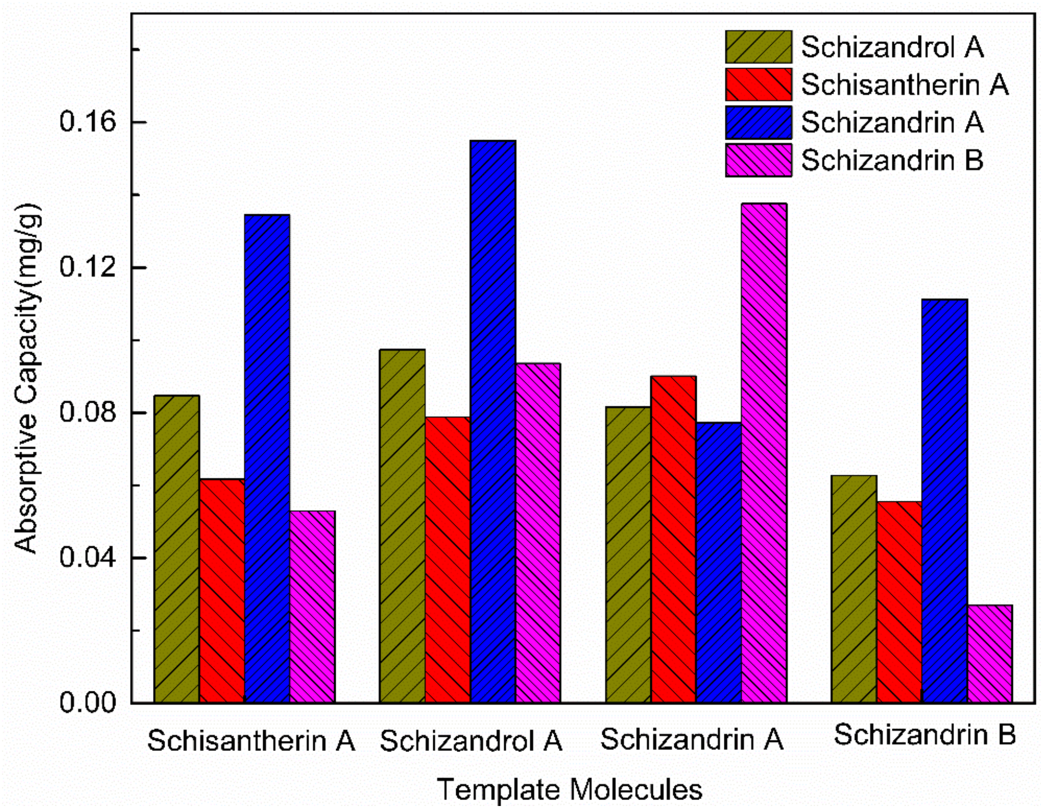

Figure S1 Template molecular screening of St-MIPs

XRD analysis of Mt-MIP and St-MIP

In this study, the crystal structure changes during polymer synthesis were analyzed by X-ray diffraction analysis. The analysis results of Fe_3O_4, Fe_3O_4@SiO_2 and Fe_3O_4@SiO_2@VTEs, Fe_3O_4@VSiO_2, NIP-1, Mt-MIP, St-MIP and NIP-2 are shown in the Figure 5. The six characteristic diffraction peaks of Fe_3O_4 nanoparticles are 30.26°, 35.6°, 43.1°, 53.78°, 57.16° and 62.78°, and the corresponding lattice surfaces are (220), (311), (400), (422), (511) and (440), respectively. These results are consistent with the magnetic crystal data of Fe_3O_4. As can be seen from the figure, the characteristic diffraction peaks in Fe_3O_4@SiO_2 and Fe_3O_4@SiO_2@VTEs, Fe_3O_4@VSiO_2, Mt-MIP, NIP-1, St-MIP and NIP-2 obtained in each step of the whole polymer synthesis are the same as the six characteristic diffraction peaks of Fe_3O_4. It indicates that the crystal lattice of Fe_3O_4 was not changed during polymer synthesis, and the reaction was carried out on the external surface of Fe_3O_4. However, the intensity of Mt-MIP, NIP-1, St-MIP and NIP-2 diffraction peaks were significantly weakened that because the external inclusion of Fe_3O_4 was gradually thickened, which affected the recognition of the lattice, and also indicated that the polymer was successfully wrapped on the support.

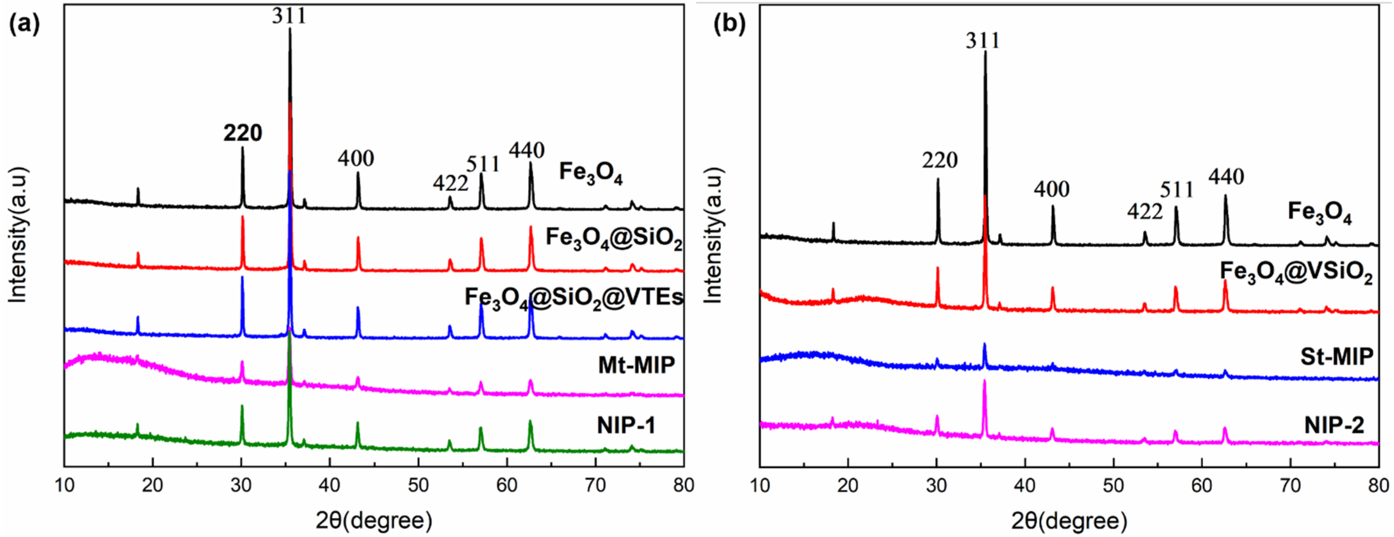

Figure S2 XRD analysis of Mt-MIP (a) and St-MIP (b).

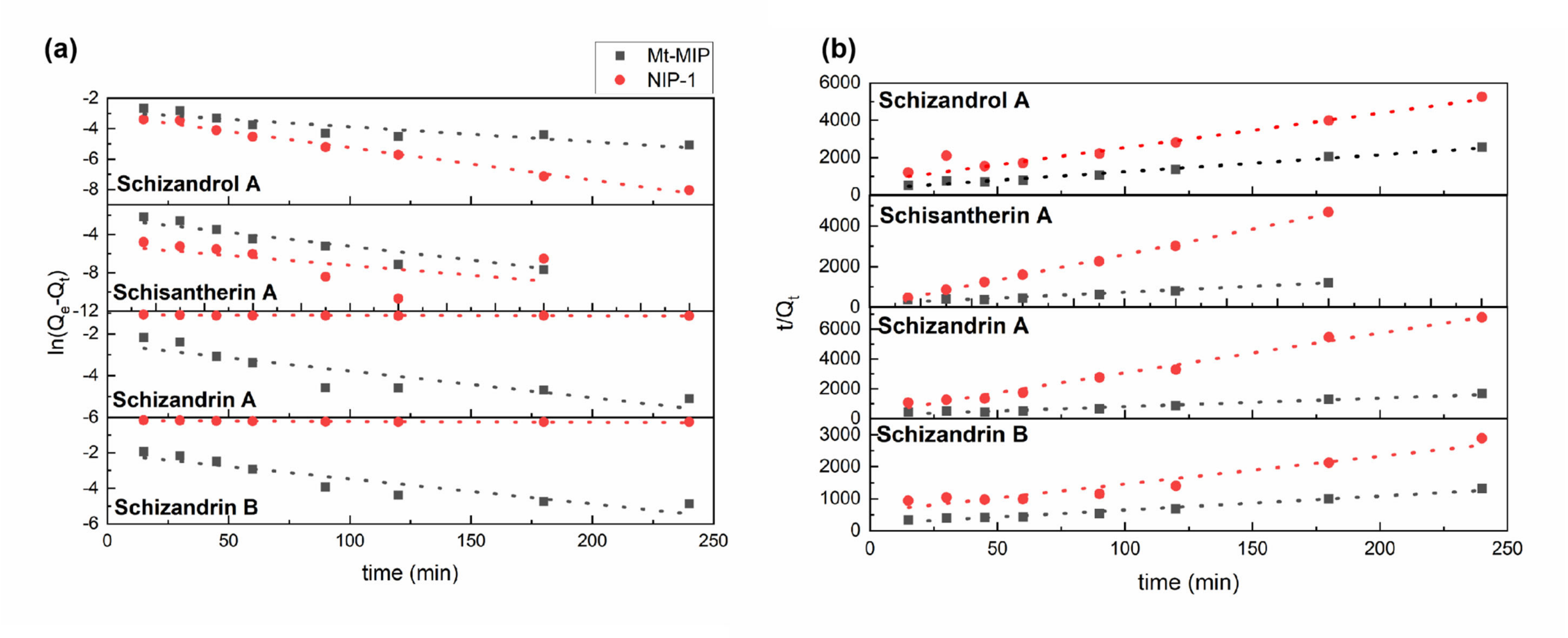

Figure S3 Kinetic fitting curves for Mt-MIP and NIP-1: (a) First-order kinetic fitting curves; (b) Second-order kinetic fitting curves.

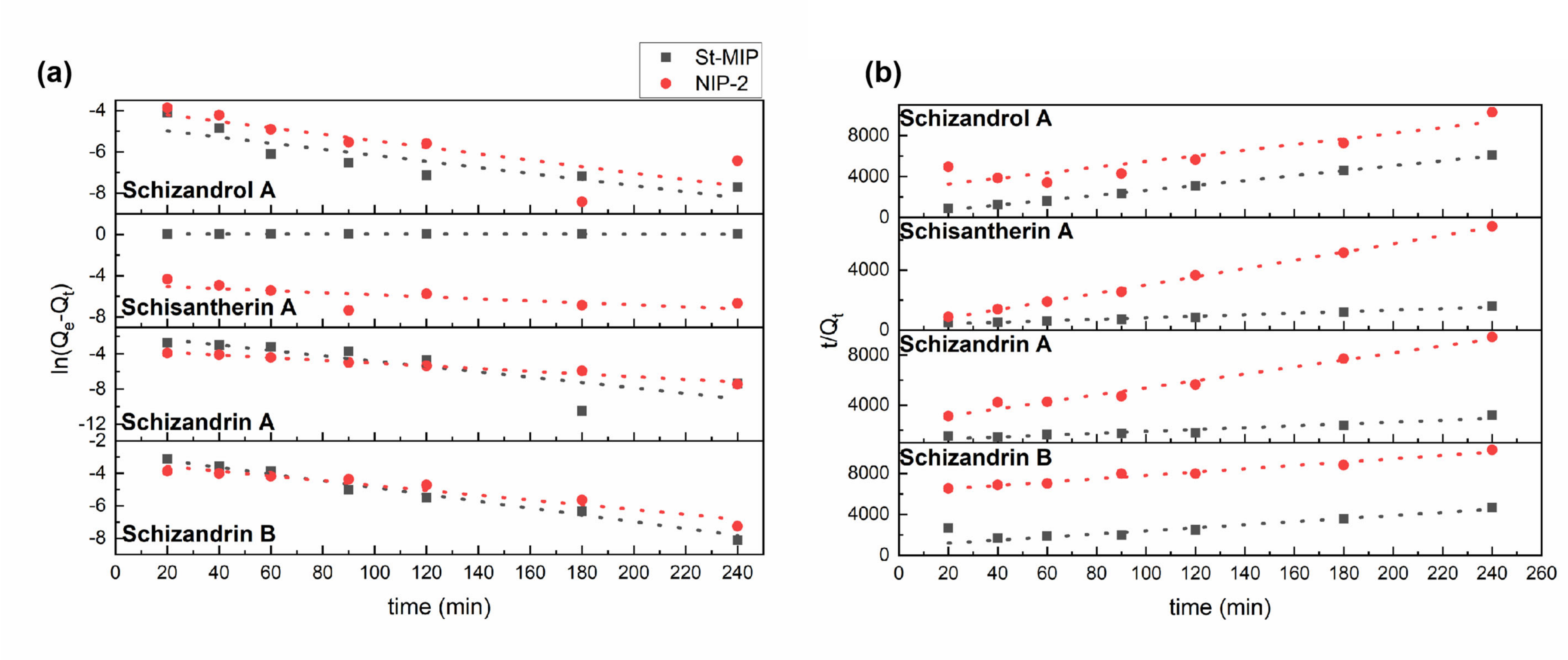

Figure S4 Kinetic fitting curves for St-MIP and NIP-2: (a) First-order kinetic fitting curves; (b) Second-order kinetic fitting curves.

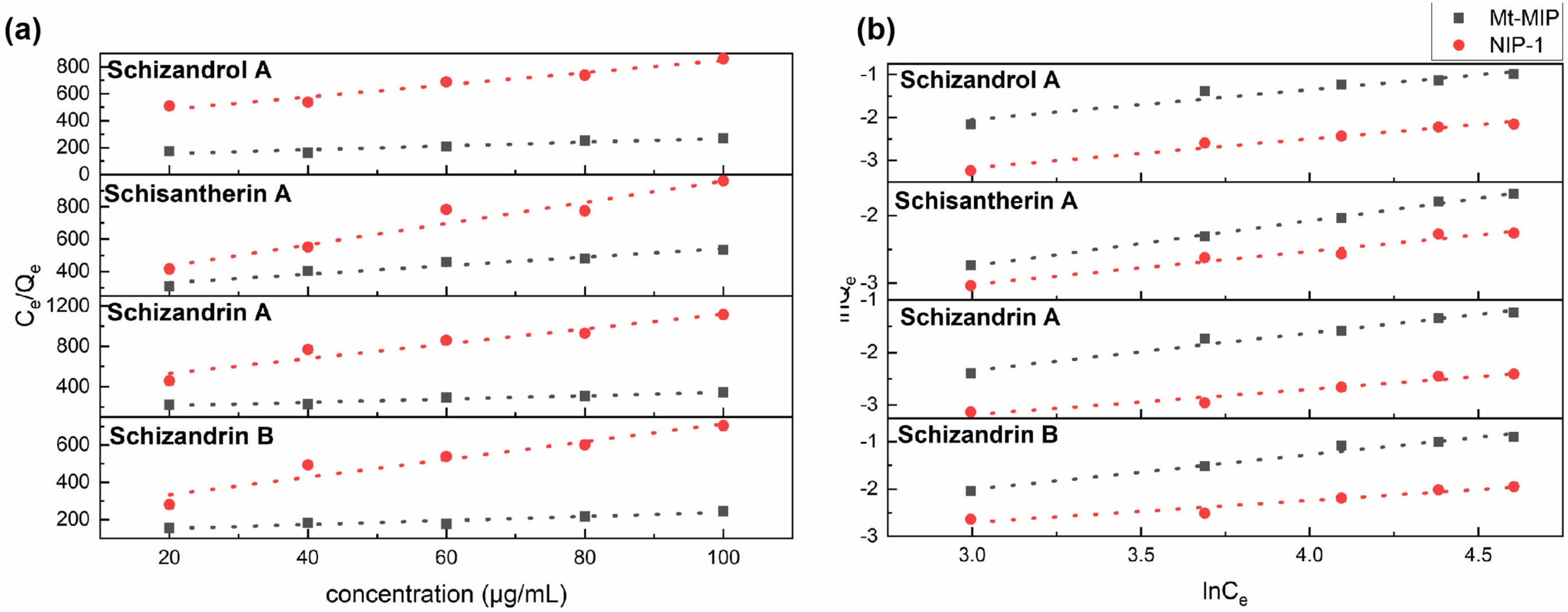

Figure S5 Adsorption isotherm fitting curves for Mt-MIP and NIP-1: (a) Langmuir model fitting curves; (b) Freundlich model fitting curves.

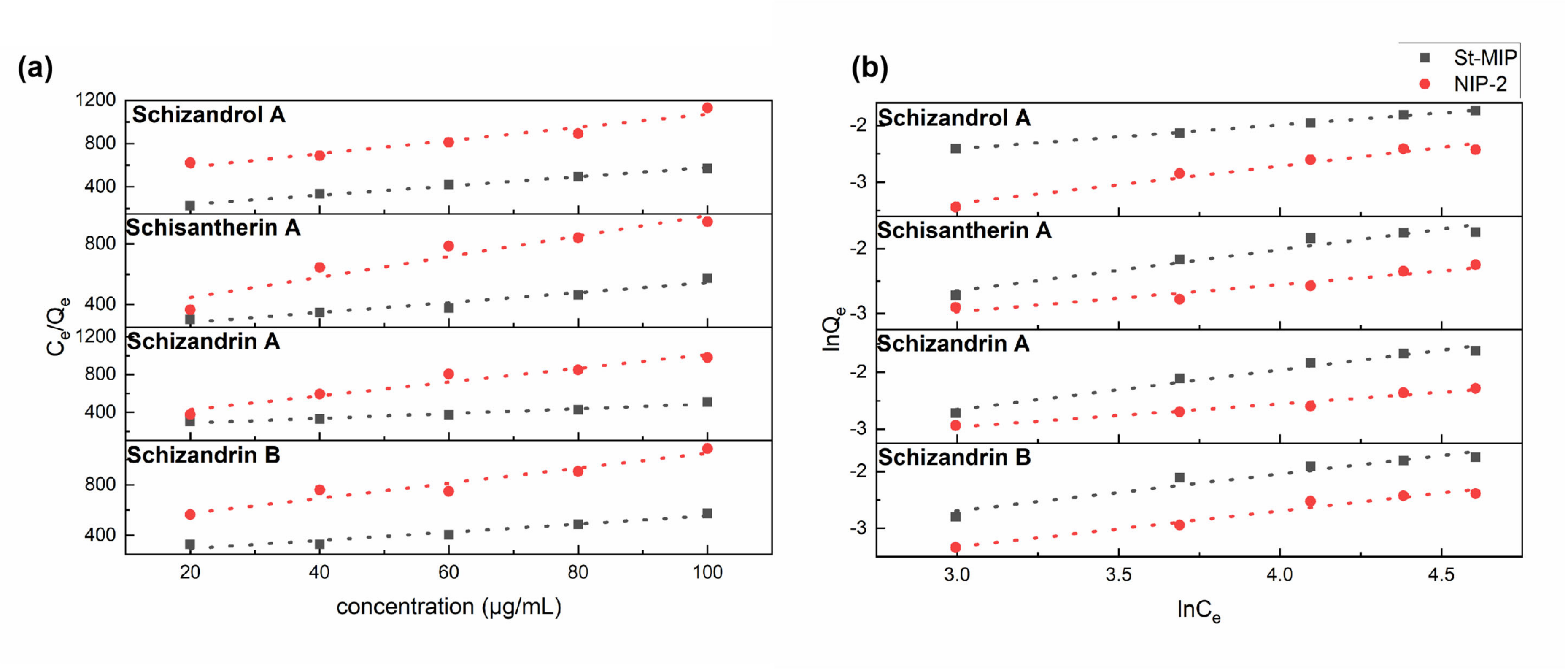

Figure S6 Adsorption isotherm fitting curves for St-MIP and NIP-2: (a) Langmuir model fitting curves; (b) Freundlich model fitting curves.

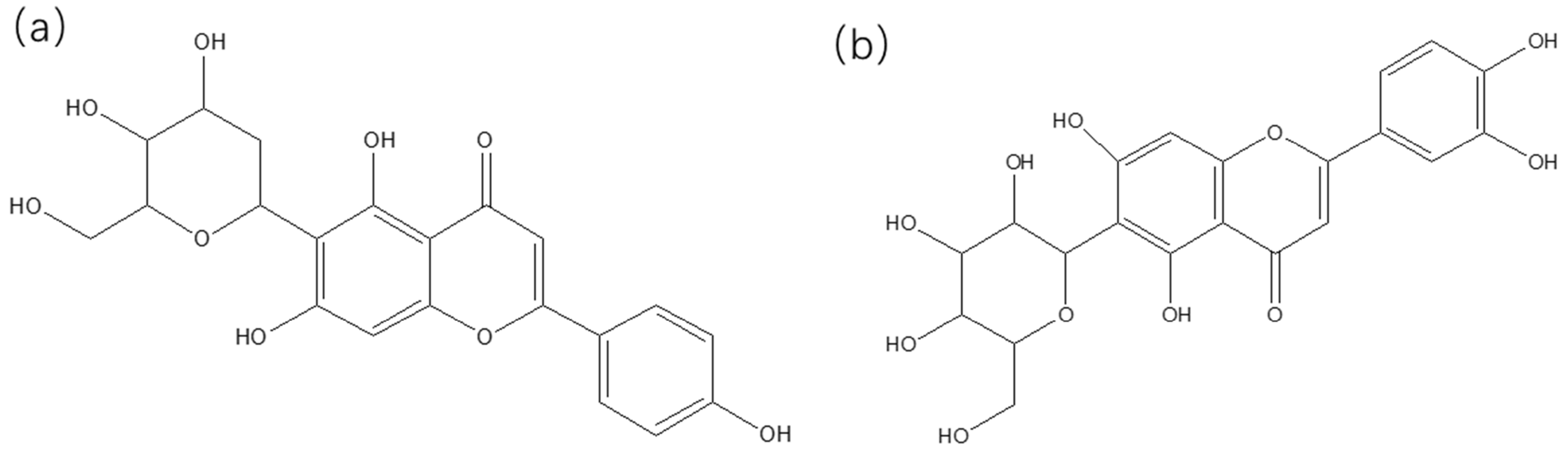

Figure S7 Chemical structure of Isovitexin (a) and Isoorientin (b)

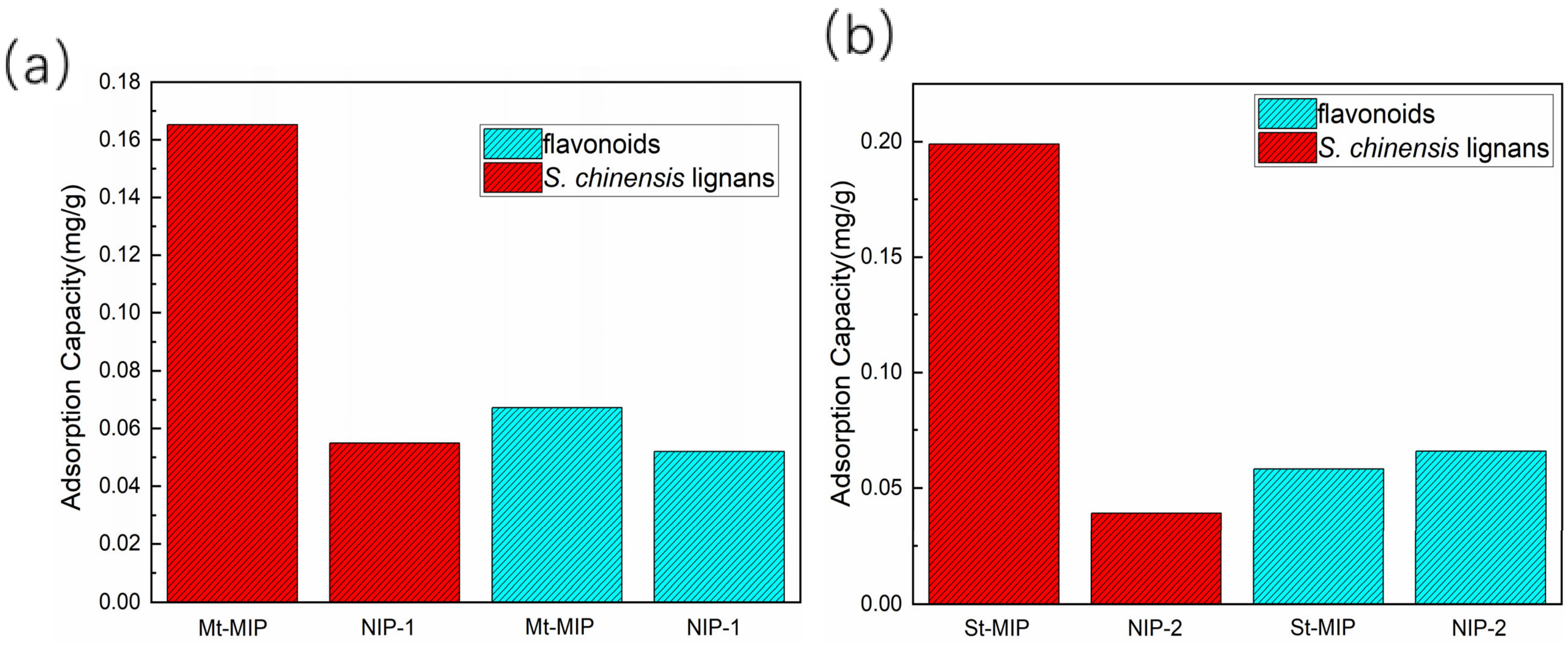

Figure S8 Selective adsorption of MIPs: adsorption capacities of *S. chinensis* lignans and flavonoids by Mt-MIP and NIP-1 (a); adsorption capacities of *S. chinensis* lignans and flavonoids by St-MIP and NIP-2 (b)

Table S1 Kinetic fitting parameters of Mt-MIP and NIP-1

		First-order kinetic				Second-order kinetic			
		K_1	Q_e	R^2	Equation	K_2	Q_e	R^2	Equation
Schizandrol A	Mt-MIP	0.0099	0.0560	0.8210	Y=-2.882-0.0099X	0.2597	0.1096	0.9842	Y=320.52+9.1257X
	NIP-1	0.0214	0.04475	0.9890	Y=-3.1067-0.0214X	0.4874	0.0543	0.9918	Y=696.86+18.43X
Schisantherin A	Mt-MIP	0.0289	0.0962	0.9118	Y=-2.3412-0.0289X	0.1901	0.1752	0.9733	Y=171.37+5.7081X
	NIP-1	0.0048	0.0057	0.0319	Y=-5.1749-0.0205X	7.5126	0.0399	0.9983	Y=83.527+25.048X
Schizandrin A	Mt-MIP	0.0128	0.0823	0.7896	Y=-2.4972-0.0128X	0.1404	0.1749	0.9689	Y=232.78+5.7161X
	NIP-1	0.0002	0.3352	0.3627	Y=-1.093-0.0002X	2.0863	0.0373	0.9884	Y=345.43+26.844X
Schizandrin B	Mt-MIP	0.0141	0.1276	0.8641	Y=-2.0591-0.0141X	0.0897	0.2294	0.9771	Y=211.83+4.3586X
	NIP-1	0.0004	0.8270	0.6624	Y=-0.19-0.0004X	0.1268	0.1153	0.9289	Y=593.23+8.6705X

Table S2 Kinetic fitting parameters of St-MIP and NIP-2

		First-order kinetic				Second-order kinetic			
		K_1	Q_e	R^2	Equation	K_2	Q_e	R^2	Equation
Schizandrol A	St-MIP	0.0147	0.0092	0.7841	Y=-4.694-0.0147X	2.3454	0.0416	0.9981	Y=246.02+24.024X
	NIP-2	0.0157	0.0205	0.6646	Y=-3.8884-0.0157X	0.2810	0.0363	0.8309	Y=2708.6+27.588X
Schisantherin A	St-MIP	0.0001	1.0486	0.6099	Y=0.0475-0.00009X	0.0816	0.1975	0.9726	Y=314.08+5.0627X
	NIP-2	0.0098	0.0078	0.4995	Y=-4.8529-0.0098X	2.9088	0.0364	0.9980	Y=259.47+27.476X
Schizandrin A	St-MIP	0.0305	0.1680	0.7024	Y=-1.7838-0.0305X	0.0471	0.1349	0.9141	Y=1167.1+7.4145X
	NIP-2	0.0154	0.2953	0.9771	Y=-3.5222-0.0154X	0.2975	0.0359	0.9816	Y=2605.3+27.84X
Schizandrin B	St-MIP	0.0210	0.0619	0.9690	Y=-2.7818-0.021X	0.2559	0.0664	0.9736	Y=887.12+15.066X
	NIP-2	0.0148	0.3787	0.9366	Y=-3.2737-0.0148X	0.0430	0.0614	0.9724	Y=6179.7+16.293X

Table S3 Adsorption isothermal model parameters of Mt-MIP and NIP-1

		Langmuir model			Freundlich model		
		k_L(mL/mg)	Q_{max}(mg/g)	R^2	k_F	n	R^2
Schizandrol A	Mt-MIP	0.1105	0.7082	0.8945	0.0160	1.4380	0.9393
	NIP-1	0.0114	0.2214	0.9642	0.0041	1.4868	0.9729
Schisantherin A	Mt-MIP	0.0095	0.3778	0.9483	0.0086	1.4952	0.9972
	NIP-1	0.0216	0.1526	0.9402	0.0113	2.0462	0.9575
Schizandrin A	Mt-MIP	0.0091	0.6115	0.9459	0.0113	1.4023	0.9785
	NIP-1	0.1910	0.1361	0.9290	0.0097	2.0644	0.9546
Schizandrin B	Mt-MIP	0.0083	0.9272	0.9019	0.0145	1.3517	0.9750
	NIP-1	0.0201	0.2100	0.9550	0.0167	2.1622	0.9714

Table S4 Adsorption isothermal model parameters of St-MIP and NIP-2

		Langmuir model			Freundlich model		
		k_L(mL/mg)	Q_{max}(mg/g)	R^2	k_F	n	R^2
Schizandrol A	St-MIP	0.0278	0.2350	0.9904	0.0254	2.3776	0.9988
	NIP-2	0.0132	0.1641	0.9427	0.0048	1.5209	0.9632
Schisantherin A	St-MIP	0.0151	0.3056	0.9369	0.0105	1.5753	0.9500
	NIP-2	0.0220	0.1473	0.9144	0.0145	2.3700	0.9282
Schizandrin A	St-MIP	0.0106	0.3970	0.9599	0.0086	1.4314	0.9743
	NIP-2	0.0257	0.1371	0.9481	0.0152	2.4492	0.9656
Schizandrin B	St-MIP	0.0142	0.3076	0.9387	0.0096	1.5375	0.9398
	NIP-2	0.0132	0.1672	0.9309	0.0054	1.5820	0.9679
